# Inoculation with *Azospirillum* sp. and *Herbaspirillum* sp. Bacteria Increases the Tolerance of Maize to Drought Stress

**DOI:** 10.3390/microorganisms5030041

**Published:** 2017-07-26

**Authors:** José Alfredo Curá, Diego Reinaldo Franz, Julián Ezequiel Filosofía, Karina Beatríz Balestrasse, Lautaro Exequiel Burgueño

**Affiliations:** Facultad de Agronomía, Cátedra de Bioquímica, Universidad de Buenos Aires, Avenida San Martín 4453, Ciudad Autónoma de Buenos Aires C1417DSE, Argentina; dfranz@agro.uba.ar (D.R.F.); filosofi@agro.uba.ar (J.E.F.); kbalestrasse@gmail.com (K.B.B.); l.burgueno@hotmail.com (L.E.B.)

**Keywords:** plant growth-promoting rhizobacteria, plant growth, plant stress, plant hormones, *ZmVP14* gene

## Abstract

Stress drought is an important abiotic factor that leads to immense losses in crop yields around the world. Strategies are urgently needed to help plants adapt to drought in order to mitigate crop losses. Here we investigated the bioprotective effects of inoculating corn grown under drought conditions with two types of plant growth-promoting rhizobacteria (PGPR), *A. brasilense*, strain SP-7, and *H. seropedicae*, strain Z-152. Plants inoculated with the bacteria were grown in a greenhouse with perlite as a substrate. Two hydric conditions were tested: normal well-watered conditions and drought conditions. Compared to control non-inoculated plants, those that were inoculated with PGPR bacteria showed a higher tolerance to the negative effects of water stress in drought conditions, with higher biomass production; higher carbon, nitrogen, and chlorophyll levels; and lower levels of abscisic acid and ethylene, which are plant hormones that affect the stress response. The oxidative stress levels of these plants were similar to those of non-inoculated plants grown in well-watered conditions, showing fewer injuries to the cell membrane. We also noted higher relative water content in the vegetal tissue and better osmoregulation in drought conditions in inoculated plants, as reflected by significantly lower proline content. Finally, we observed lower gene expression of *ZmVP14* in the inoculated plants; notably, *ZmVP14* is involved in the biosynthesis of abscisic acid. Taken together, these results demonstrate that these bacteria could be used to help plants cope with the negative effects of drought stress conditions.

## 1. Introduction

Corn (*Zea mays* L.) farming is one of the most important and extensive farming systems in the world because of the myriad products derived from this plant. In 2013, a total of 1,018,111,958 tons of corn were produced globally [1]. Drought is a major abiotic stress factor that affects crop yield. Daryanto et al. [2] collected data from peer-reviewed publications between 1980 and 2015 that examined maize and wheat yield responses to drought using field experiments and concluded that the maize yield reduction was 39.3%. The loss in crop yield during drought depends on the phenological stage of the crops and on the severity of the hydric deficit [3]. In coming years, global warming is predicted to increase the severity and the frequency of drought. According to Food and Agriculture Organization of the United Nations (FAO), the Food and Agriculture Organization of the United Nations, agriculture must adapt to the effects of global warming and improve crop resilience in order for food production to meet the food needs of our increasing population. Accordingly, the production of major crops will have to shift to marginal areas, some with hydric deficits [4].

The reactions of plants to drought stress are complex. Factors that affect the response include the environment, plant genotype, plant development stage, and the severity and duration of the stress [5]. Morpho-physiological and biochemical characteristics related to drought stress include leaf senescence, decreases in the foliar area and the chlorophyll content, root elongation, decreases in the relative water content (RWC), increases in reactive oxygen species (ROS) production [6] and nitrate assimilation [7]. In addition to the many physiological and cellular changes, there are also changes in many genes and gene products in response to drought stress that occur at the transcriptional, post-transcriptional, and translational levels [5]. Some responses to water stress are related to changes in ion flux, stomatal closing, the production of osmoprotectant metabolites, and alterations in plant growth patterns [8]. Plants regulate their hydric state via stomatal closing, a process that is influenced by the hormone abscisic acid (ABA), which is synthesized mainly in the leaves. ABA synthesis is stimulated by dehydration conditions, and ABA plays an important role in the response to drought stress [9,10,11]. Hydric deficit also decreases photosynthesis, leading to stomatal closing and to decreases in the intercellular CO_2_ concentration [12]. 

The enzyme 9-*cis*-epoxycarotenoid dioxygenase (NCED), which is found in chloroplasts, acts to produce xanthoxin, and this reaction is considered the first step in ABA synthesis. NCED belongs to a multigene family in *Zea mays* L., and the VP14 gene in corn was the first cloned NCED gene [13,14]. NCED is the most important regulating enzyme in ABA syntheses because its expression correlates with endogenous ABA content and its overexpression leads to important ABA accumulation [15]. Tan et al. [16] revealed the role of the VP14 gene in ABA synthesis, and reported that the VP14 protein is expressed as a response to drought stress and concluded that VP14 encodes a dioxygenase that is responsible for the oxidative conversion of neoxanthin to xanthoxin. This is one step in the ABA synthesis chain; xanthoxin is subsequently converted into an ABA aldehyde by a short-chain alcohol dehydrogenase (ABA2 in *Arabidopsis*).

In addition to ABA, ethylene is another hormone that is associated with the response of plants to stress. Ethylene levels regulate many processes involved in the growth and development of plants, and ethylene biosynthesis is regulated by both biotic and abiotic stress [17]. During stress conditions, endogenous ethylene in the plant reduces the growth of roots and stems. 1-Aminocyclopropane-1-carboxylic (ACC) is a precursor of ethylene synthesis in plants. Glick [18] showed that plant ACC is broken down by the bacterial enzyme ACC deaminase, which converts ACC into a source of nitrogen and energy. Under stressful conditions, plants produce reactive oxygen species (ROS) such as hydrogen peroxide, superoxide anion, and hydroxyl radicals [19]. Oxidative stress is caused by an imbalance between ROS productivity and the ability of a biological system to detoxify the reactive intermediates or to repair the resulting damage [20]. The damage caused by ROS can be assessed by studying the lipid peroxidation of cellular membranes, with malondialdehyde (MDA) used as a marker of this process [21].

Osmotic regulation refers to the ability of a plant to accumulate solutes as a response to a hydric deficit, a phenomenon that can totally or partially preserve turgor pressure [22]. Importantly, ABA induces the synthesis of several solutes called osmolytes. These small, uncharged, soluble molecules, which include proline, glycine betaine, polyamines, and melatonin, do not affect cellular function directly. These solutes decrease the hydric potential of cells by trapping water molecules or by retaining the water molecules they are already associated with. Compatible solutes can increase the stability and integrity of membranes and proteins, preventing or lessening cellular damage. During hydric deficit conditions, it was observed that the proline concentration could increase to double its normal level, showing clear osmoregulation [23].

Changes in factors that affect plant growth and development can lead to losses in crop yields, highlighting the importance of developing new and alternative technologies to achieve sustainable farming conditions. One such technology is the use of plant growth-promoting rhizobacteria (PGPR) that reduce the harmful effects of abiotic stress. 

There is a large group of PGPR bacteria that colonize the rhizosphere and interact with plants, helping them to grow in both direct and indirect ways [24,25,26,27]*.* For example, PGPR produce some metabolites that can be used by plants as growth regulators, such as auxins, gibberellins, and cytokinins [28,29,30,31]. PGPR also influence the biological fixation of nitrogen [28,31,32] and nitrate assimilation [32,33,34], and they can increase the availability of nutrients such as phosphorus by solubilizing insoluble phosphates [35]. PGPR also produce some antagonistic metabolites that negatively affect microorganisms that are detrimental to the plant; such metabolites include antibiotics [36,37], siderophores [38], and chitinolytic enzymes and glucanases [39,40].

*Azospirillum* sp. are among the most studied PGPR [41,42,43,44]. These bacteria have been used successfully as inoculants in different crop and agro-ecological conditions, and they help increase crop production efficiency [45]. Many groups have reported that *Herbaspirillum* sp. bacteria can help the growth and productivity of some economically important crops, such as rice, corn, and sugar cane [46,47,48,49]. Some studies have shown that using PGPR can lead to a higher tolerance to abiotic stress conditions, including drought and salinity [50,51,52,53,54,55,56,57,58]. However, little is known about the molecular processes involved in the interaction of plants and bacteria in drought conditions. 

## 2. Materials and Methods

### 2.1. Experimental Design and Statistical Analysis

We designed a factorial experiment with two factors. The first factor, bacteria, had three levels: (1) control seeds treated with sterile NFb medium [59]; (2) seeds inoculated with *Azospirillum brasilense*, strain SP-7 (ATCC 29729™); and (3) seeds inoculated with *Herbaspirillum seropedicae*, strain Z-152 (ATCC 35894™). The second factor, watering, had two levels: (1) watering once every 24 h, called the well-watered (WW) condition; (2) watering once every 96 h, called the drought (D) condition. The experiment involved six treatments, each composed of three replicates. Each experimental unit or replicate had 36 samples. The data were analyzed using analysis of variance (ANOVA), including the interaction between the bacteria and watering factors as a source of variation. We also performed the Tukey test of multiple comparisons using the statistical software InfoStat [60].

### 2.2. Bacterial Growth and Inoculation

We used NFb medium [59] to grow strain *A. brasilense*, and JNFb medium [61] to grow strain *H. seropedicae*. To inoculate the seeds, we used a concentration of 1.6 × 10^9^ CFU mL^−1^ (colony-forming units per milliliter) of *A. brasilense* and 1.8 × 10^9^ CFU mL^−1^ of *H. seropedicae* in a volume of 50 mL of the appropriate medium. Before inoculation, the seeds were rinsed for 15 min in a solution of 30% *v*/*v* of commercial bleach, 70% sterile distilled water, and 100 μL/L of Triton X-100. The seeds were then rinsed three times for 10 min/rinse in sterile water. For inoculation, the seeds were placed in bacterial medium for 24 h.

### 2.3. Plant Growth Conditions

This experiment was conducted in a greenhouse with temperatures between 25 °C and 30 °C, a 16 h/8 h light/dark cycle, and 50–70% humidity. We used a 36-well tray (Speedling Incorporated, Nipomo, CA, USA). Each well had a volume of 250 mL, and sterile perlite was used as the substrate. Each tray was treated the same way in order to establish the experimental unit. After the seeds were inoculated, we placed one seed in each well. We watered each well with 40 mL of Murashige & Skoog Salt Mixture medium [62] at a concentration of 0.20 in distilled water. During the first 8 days of the test, all plants were watered once every 48 h for every treatment condition. Starting on day 8, the plants were watered once every 48 h in the WW condition and once every 96 h in the D condition. The experiment was complete 20 days after sowing (DAS).

### 2.4. Measures

#### 2.4.1. Symbiotic Development

Once the experiment was complete, we took samples of the roots of plants that were inoculated with the bacterial strains. We then cleaned the root surface with 25 mL of the solution 30% *v*/*v* of commercial bleach, 70% sterile distilled water, and 100 μL/L of Triton X-100. Using a mortar, we extracted a homogenate from which we prepared serial dilutions. The CFU of each strain was determined after 5 days of incubation at 30 °C in plates with solid Congo red medium [63], using colony shape and color as a way to confirm the identities of each strain.

#### 2.4.2. Total Biomass (TB) Production

In order to determine the dry weight of the TB, we collected six random samples (entire plants) from each treatment condition 20 DAS. The samples were placed in an 80 °C oven for 48 h. When the sample was dry, we determined its weight.

#### 2.4.3. Total Carbon (TC) and Total Nitrogen (TN) Content

In order to analyze the TC and the TN, we collected three random samples (entire plants) from each treatment condition 20 DAS. We used a modified Walkley-Black method [64] for TC determination, and we used the Kjeldahl method [65] for TN determination.

#### 2.4.4. Total Chlorophyll (TChl) Content

The TChl content was determined using the method described by Inskeep and Bloom [66]. We collected three random samples (leaves from three plants) from each treatment condition 20 DAS. Disc-shaped samples (1-cm in diameter) were taken from the leaves, avoiding the central nervation. The weight of four discs per sample was taken. Then the four discs were placed in Eppendorf tubes containing 2 mL of dimethylformamide (DMF). The tubes were covered with aluminum foil and stored in a refrigerator at 4 °C for 4 days. The absorbance of each sample was then read at 647, 652, and 664 nm using a spectrophotometer. Chlorophyll content in mg total chlorophyll per g fresh weight was expressed.

#### 2.4.5. ABA and Ethylene Content

In order to determine the ABA content, we collected three random samples (entire plants) from each treatment condition 20 DAS. Then we performed an extraction using the methods of Kelen et al. [67] and Iriti et al. [68]. For analysis, we used an Agilent 1100 Series HPLC with a Zorbax Eclipse XDB C18 column (150 × 4.6 mm; 5 μm particle size). The mobile phase was MeOH:H_2_O 70:30, pH 4.0; flux was 0.5 mL/min; and UV detection was at 265 nm. The injection volume was 20 μL, and the ABA retention time was 3.9 min. 

In order to determine the ethylene content, we randomly collected three leaves per treatment condition 20 DAS. These leaves were placed in 10-mL jars with 1 mL of the regulator solution (50 mM Na_2_HPO_4_/NaH_2_PO_4_, pH 6.8). The jars were sealed with a rubber septum and incubated for 24 h at 25 °C in the dark. Ethylene production was measured using gas chromatography [34].

#### 2.4.6. Relative Water Content (RWC)

In order to determine the RWC, we collected six random samples (the last leaf completely enlarged) from each treatment condition 20 DAS, to assess the fresh weight. We determined the weight of the completely turgid sample and the dry weight according to the methods of Naveed et al. [55]. To evaluate the RWC we used the following equation [69]:

(1) $$\mathrm{RWC}\;\left(\%\right)\;=\frac{\mathrm{Fresh}\;\mathrm{weight}\;-\;\mathrm{Dry}\;\mathrm{weight}}{\mathrm{Completely}\;\mathrm{turgid}\;\mathrm{weight}\;-\;\mathrm{Dry}\;\mathrm{weight}}\times \;100$$

#### 2.4.7. Malondialdehyde (MDA) Content

To determine the MDA content, we collected four random samples (entire plants) from each treatment condition 20 DAS. These fresh samples were homogenized in a 20% *p*/*v* trichloroacetic acid (TCA) solution and centrifuged at 3500× *g* for 20 min. A 1-mL aliquot of the supernatant was added to 1 mL of 20% TCA solution plus 0.5% (*p*/*v*) thiobarbituric acid and 100 μL of butylated hydroxytoluene (from a 4% solution in ethanol). This mixture was heated at 95 °C for 30 min, cooled on ice, and centrifuged at 10,000× *g* for 15 min. We determined the absorbance of an aliquot of the supernatant at 532 nm and subtracted the value for non-specific absorption at 600 nm. The concentration of thiobarbituric acid reactive substances (TBARS) was calculated using an extinction coefficient of 155 mM^−1^ cm^−1^ as described by Heath and Packer [70].

#### 2.4.8. Proline Content

To determine the proline content, we collected four random samples (entire plants) from each treatment condition 20 DAS. With a cold (4 °C) mortar, we ground 0.5 g of fresh tissue and homogenized it in 5 mL of 3% (*p*/*v*) sulfosalicylic acid to precipitate the proteins. The homogenized sample was filtered through Whatman grade 2 filter paper, and 2 mL of the filtered sample or the proline standard was placed in a test tube and was mixed with 2 mL of glacial acetic acid and 2 mL of acid ninhydrin. The solution was shaken and incubated at 100 °C for 1 h, creating a colored complex. The reaction was stopped by placing the sample in an ice bath, then 4 mL of toluene was added to each test tube and each sample was mixed using a vortex mixer for 15–20 s. Finally, the two phases were allowed to separate. The organic (toluene) phase was recovered, and measurements were performed at 520 nm using a spectrophotometer [71].

#### 2.4.9. Differential Expression of the *ZmVP14* Gene

The *ZmVP14* gene was amplified and quantified using real time PCR and SYBR^®^ Green to detect the product. Random samples were collected 20 DAS. The primers used to amplify the *ZmVP14* gene were as follows: forward 5′-TCCACGACTTCGCCATCACC-3′ and reverse 5′-CGTCTTCTCCTTGTCCAGCACC-3′. The products were quantified relative to the control treatment (WW) using the 2^−ΔΔ*C_T_*^ method [72]. Expression of the endogenous actin gene served as a control using the following primers: forward 5′-TCCTGACACTGAAGTACCCGATTG-3′ and reverse 5′-CGTTGTAGAAGGTGTGATGCCAGTT-3′. We used the following amplification conditions: 50 °C for 2 min; 95 °C for 10 min; (95 °C for 15 s and 54 °C for 1 min) × 45 cycles. We used a dissociation curve (melting) to make sure there was no nonspecific amplification.

## 3. Results

### 3.1. Symbiotic Development

In the WW condition, the *A. brasilense* concentration was 9.2 × 10^5^ CFU mL^−1^ and the *H. seropedicae* concentration was 6.3 × 10^5^ CFU mL^−1^*.* In the D condition, the *A. brasilense* concentration was 2.5 × 10^4^ CFU mL^−1^ and the *H. seropedicae* concentration was 4.2 × 10^4^ CFU mL^−1^*.*

### 3.2. TB Production

**Figure 1:**
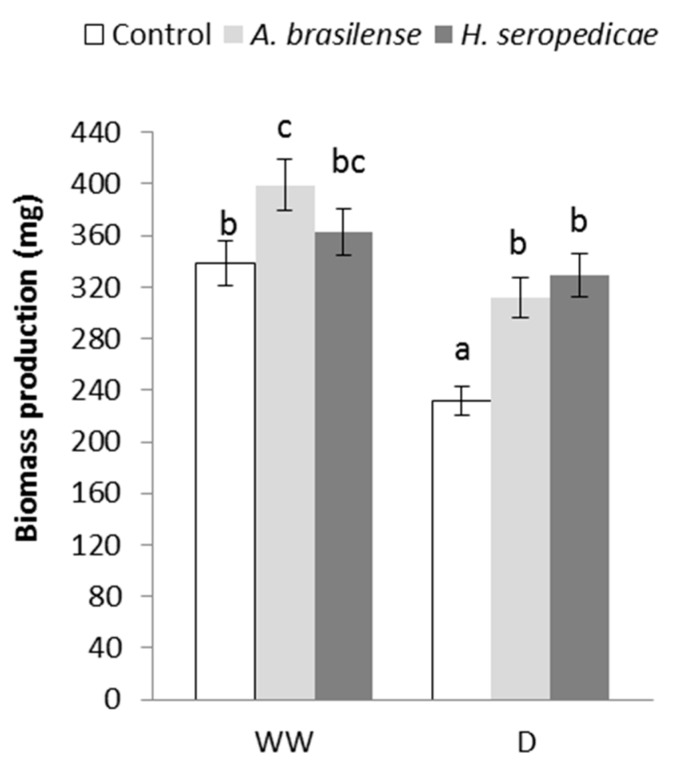
Total biomass production 20 days after sowing (DAS). Data are shown as means ± standard deviations (SD) (*n* = 6). Means with common letters are not significantly different (*p* > 0.05). The plants were cultivated under well-watered (WW) or drought (D) conditions. The inoculation * drought interaction *p* = 0.0213.

Figure 1 shows the TB production results. In the D condition, plants inoculated with *H. seropedicae* showed 29.5% greater TB production than control plants, and plants inoculated with *A. brasilense* showed 26% greater TB production than control plants. In the WW condition, plants inoculated with *A. brasilense* showed 15% greater TB production than control plants.

### 3.3. TC and TN Content

**Figure 2:**
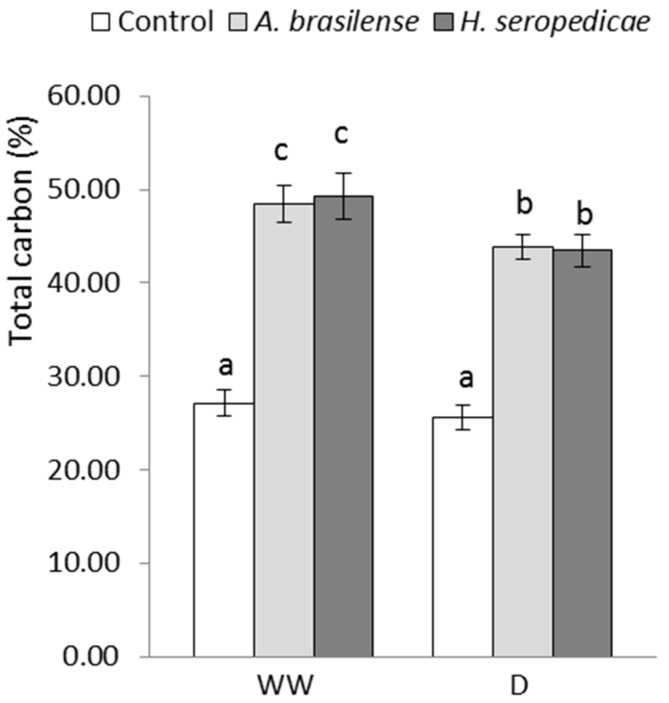
Percentage of total carbon content 20 DAS. Data are shown as means ± SD (*n* = 3). Means with common letters are not significantly different (*p* > 0.05). The plants were cultivated under WW or D conditions. The inoculation * drought interaction *p* = 0.0473.

**Figure 3:**
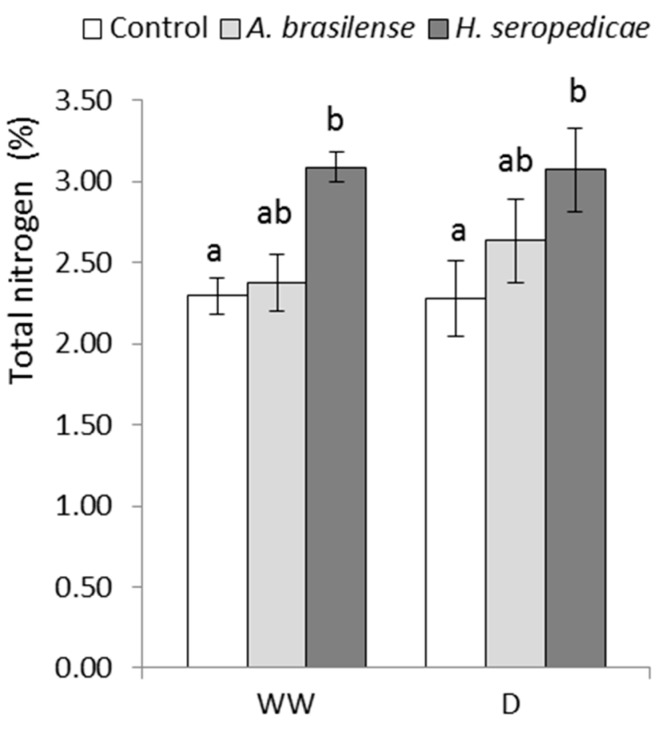
Percentage of total nitrogen content 20 DAS. Data are shown as means ± SD (*n* = 3). Means with common letters are not significantly different (*p* > 0.05). The plants were cultivated under WW or D conditions. The inoculation * drought interaction *p* = 0.0482.

Figure 2 shows the TC content results. In the D condition, plants inoculated with either PGPR had significantly (41%) more TC than control plants (*p* ≤ 0.05). In the WW condition, plants inoculated with either PGPR produced approximately 45% more TC than the control plants. There were significant differences in the TN content according to the inoculated bacterial strain, as shown in Figure 3. In both the D and the WW conditions, plants inoculated with *H. seropedicae* produced 26% more TN than the controls (*p* ≤ 0.05).

### 3.4. TChl Content

**Figure 4:**
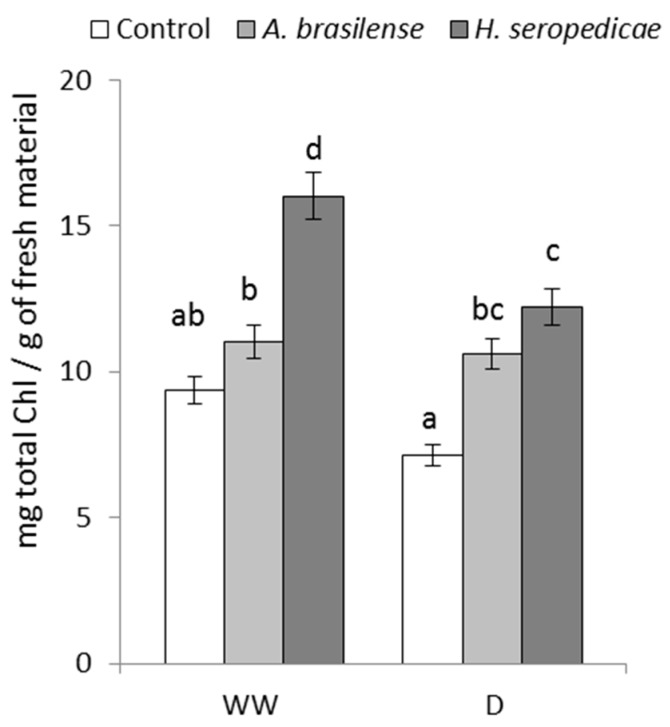
Total chlorophyll content 20 DAS. Data are shown as means ± SD (*n* = 3). Means with common letters are not significantly different (*p* > 0.05). The plants were cultivated under WW or D conditions. The inoculation * drought interaction *p* = 0.0273.

Figure 4 shows the TChl content results. In the D condition, plants inoculated with either PGPR had significantly more TChl than control plants (*p* ≤ 0.05). Specifically, plants inoculated with *H. seropedicae* produced 41.4% more TChl than controls, and plants inoculated with *A. brasilense* produced 33% more. In the WW condition, plants inoculated with *H. seropedicae* produced 41% more TChl than control plants (*p* ≤ 0.05).

### 3.5. ABA and Ethylene Content

**Figure 5:**
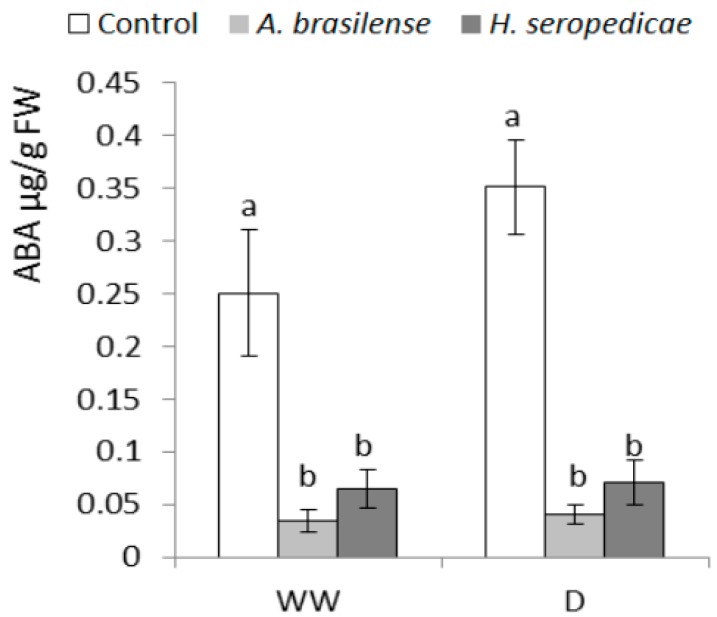
Abscisic acid (ABA) content 20 DAS. Data are shown as means ± SD (*n* = 24). Means with common letters are not significantly different (*p* > 0.05). The plants were cultivated under WW or D conditions. The inoculation * drought interaction *p* = 0.0908.

**Figure 6:**
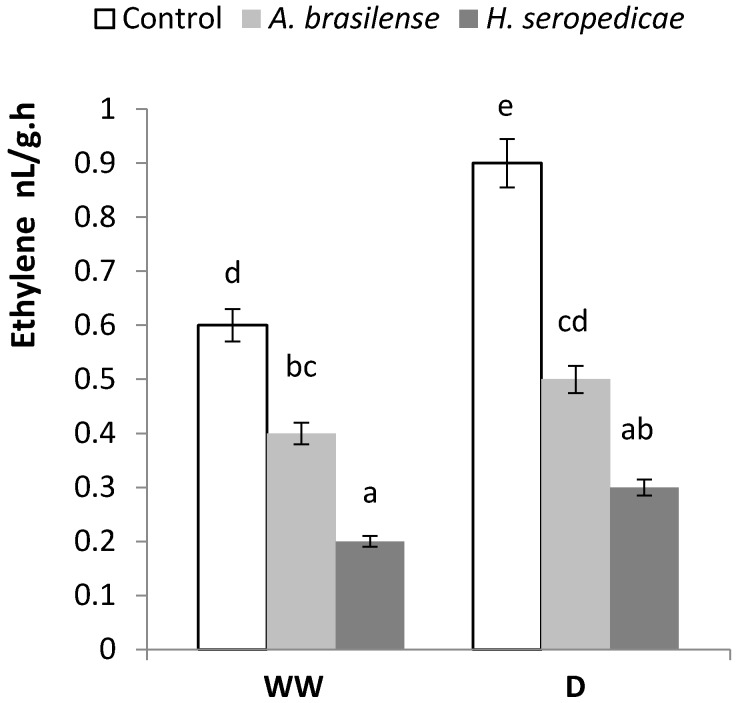
Ethylene content 20 DAS. Data are shown as means ± SD (*n* = 3). Means with common letters are not significantly different (*p* > 0.05). The plants were cultivated under WW or D conditions. The inoculation * drought interaction *p* = 0.0297.

Figure 5 shows the ABA content results. In both the WW and D conditions, control plants had significantly higher ABA content than the PGPR-inoculated plants (*p* ≤ 0.05). In the inoculated plants, the ABA content did not differ significantly in D versus WW conditions. In control plants, the ABA content was 30% higher in plants in D conditions versus WW conditions, but this difference was not significant. Figure 6 shows the ethylene content results. In both the WW and D conditions, control plants showed higher ethylene content than inoculated plants, and the ethylene content was not significantly different in the inoculated plants in D versus WW conditions (inoculation * drought interaction *p* = 0.0297).

As can be seen in Figure 6, the ethylene production is increased in non-inoculated plants under drought conditions.

### 3.6. RWC

Table 1 shows the RWC (%) of the plants according to inoculation status and hydric conditions. The RWC was higher in plants inoculated with either PGPR in both WW and D conditions. In the WW condition, plants inoculated with *A. brasilense* had the highest RWC (10% higher) versus control plants. In the D condition, plants inoculated with *H. seropedicae* had the highest RWC (5.5% higher) versus control plants. 

### 3.7. Proline Content

**Figure 7:**
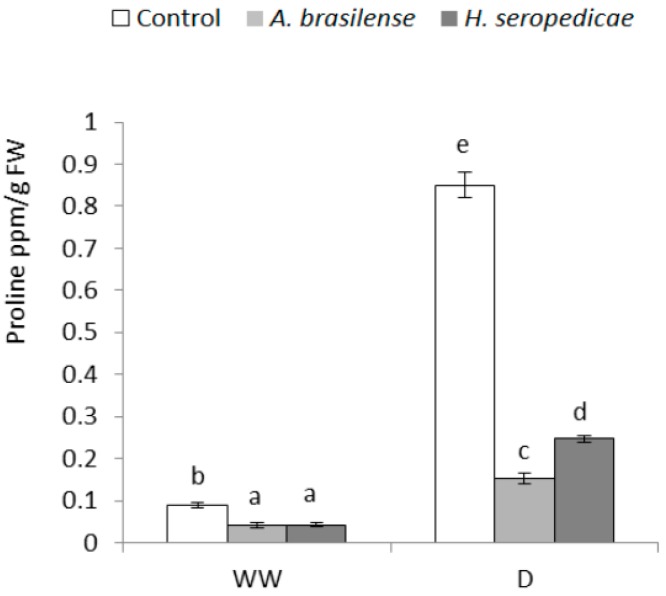
Proline concentration 20 DAS. Data are shown as means ± SD (*n* = 24). Means with common letters are not significantly different (*p* > 0.05). The plants were cultivated under WW or D conditions. The inoculation * drought interaction *p* < 0.0001.

Proline is an indicator of osmoregulation, and was measured during the experiment; the results are indicated in Figure 7. In the WW condition, control plants had a higher proline concentration than plants inoculated with either PGPR. In the D condition, the proline levels increased significantly over time regardless of inoculation status, but control plants had the highest concentration of proline relative to the WW condition (eight-fold more proline than control plants in the WW condition). In the same condition hydric (D), the plants inoculated with *H. seropedicae* showed the greatest increase (four-fold) in proline over time, while those inoculated with *A. brasilense* showed a two-fold increase in the proline level (*p* ≤ 0.05). 

### 3.8. MDA Content

**Figure 8:**
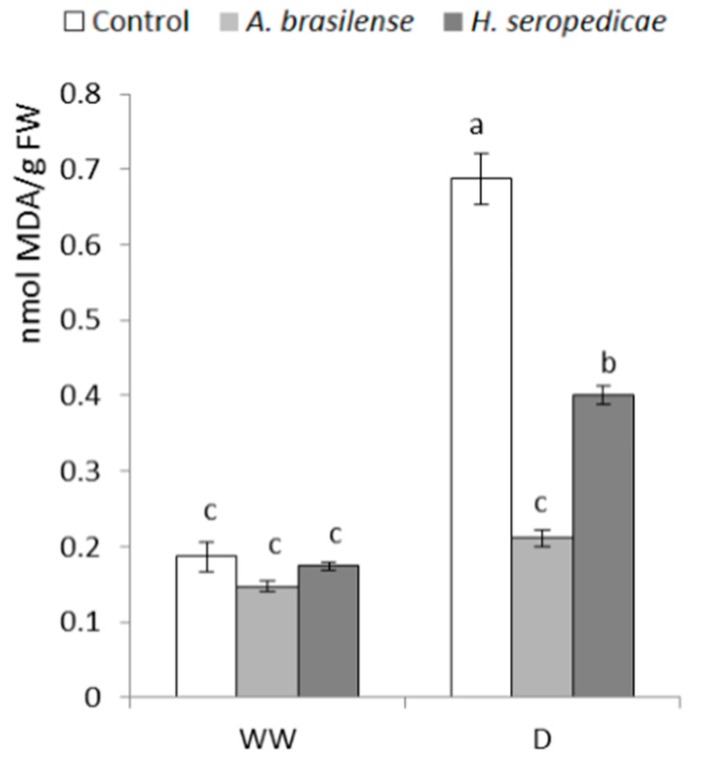
Malondialdehyde (MDA) concentration 20 DAS. Data are shown as means ± SD (*n* = 24). Means with common letters are not significantly different (*p* > 0.05). The plants were cultivated under WW or D conditions. The inoculation * drought interaction *p* < 0.0001.

MDA was quantified in order to assess the integrity of the cellular membranes, since MDA acts as a lipid peroxidation indicator. Figure 8 shows the MDA content results 20 DAS. In the D condition, plants inoculated with *A. brasilense* showed better membrane stability than plants inoculated with *H. seropedicae* and control plants. In the D condition, plants inoculated with *H. seropedicae* showed a two-fold increase in MDA content over time, while control plants showed a >300% increase in MDA content. In the D condition, plants inoculated with *A. brasilense* showed a ~30% increase in MDA content, which was not significantly different than control plants in the WW condition (*p* ≤ 0.05).

### 3.9. Expression of the ZmVP14 Gene

**Figure 9:**
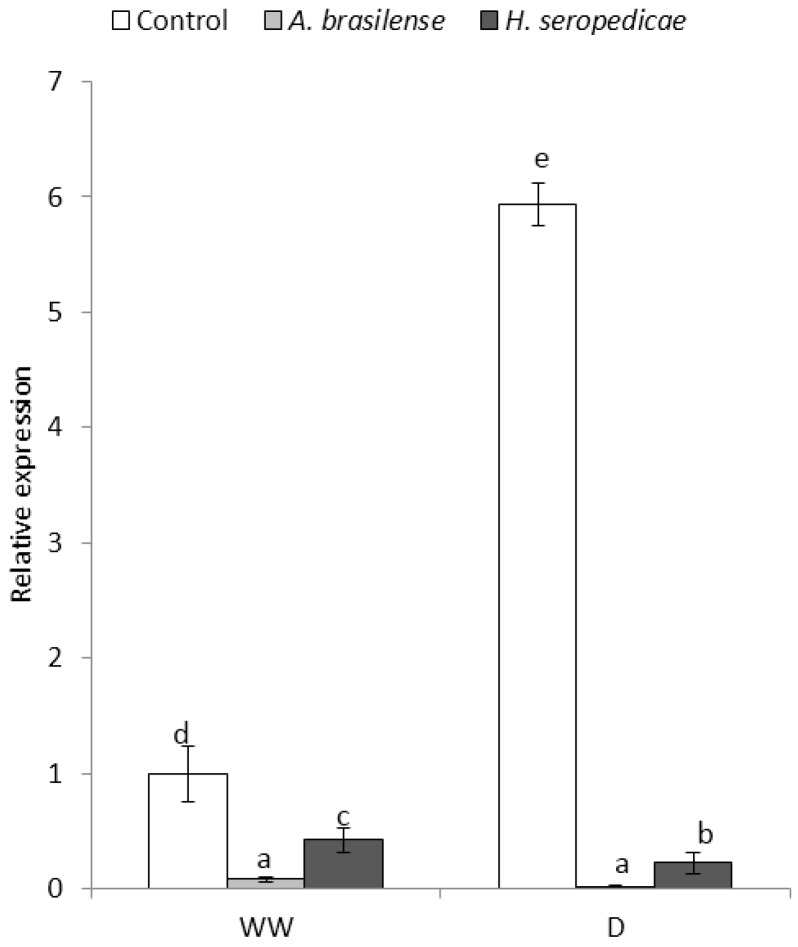
Relative expression of the *ZmVP14* gene 20 DAS. Data are shown as means (*n* = 5). Means with common letters are not significantly different (*p* > 0.05). The plants were cultivated under WW or D conditions. The inoculation * drought interaction *p* < 0.0001.

Figure 9 shows the results of the *ZmVP14* gene expression analysis. The gene expression level was considered to be 1 in control plants in the WW condition. In the WW condition, *ZmVP14* gene expression was higher in control plants than in PGPR-inoculated plants. In control plants, *ZmVP14* gene expression was almost five-fold higher in the D condition than in the WW condition. *ZmVP14* gene expression was almost undetectable in plants inoculated with *A. brasilense* in both water conditions.

## 4. Discussion

Determination of the CFU mL^−1^ in the root samples showed that *A. brasilense* and *H. seropedicae* effectively colonized the corn seedlings in plants grown in both the WW and D conditions. Thus, bacterial growth was not significantly affected by the D condition, and our results were similar to those obtained by Ruíz-Sánchez et al. [53] with arbuscular micorrhiza and *Azospirillum* sp. in rice, by Naveed et al. [55] with other PGPR (*Burkholderia* sp. and *Enterobacter* sp.) in corn, and by Cohen et al. [56] with *Azospirillum* sp. in *Arabidopsis thaliana*. 

The TB results (Figure 1) showed that the bacteria promoted vegetal growth in both hydric conditions, but the differences in growth in inoculated versus control plants were greater in D conditions (*p* = 0.0213). These results may be related to a lower level of stress in the inoculated plants, which showed higher TB. Our results were similar to those obtained by Ruíz-Sánchez et al. [53], Naveed et al. [55], Cohen et al. [56], and Tiwari et al. [67], using *Pseudomonas* sp. in *Cicer arietinum* L. There were other interesting results related to TB production that are shown in Figure 2, Figure 3 and Figure 4, in terms of the TC, TN, and TChl contents. Specifically, when TB was greater, TC (*p* = 0.0473) and TN (*p* = 0.0482) were also higher regardless of the inoculation status or hydric condition. These results may be related to a lower level of stress in inoculated plants, which showed lower ABA content. This allowed the stomata to stay open, even in drought conditions, and also allowed better fixation of atmospheric CO_2_ in the carbon compounds used in biomass production. These results are in accordance with those of Naveed et al. [55], who showed higher CO_2_ assimilation levels, stomatal conductance, and transpiration rate in inoculated corn plants in a drought condition. On the other hand, the higher nitrogen content of inoculated plants may be related to the nitrogen that is provided by the PGPR, which is the result of the biological fixation of atmospheric N_2_ [47]. Regarding the TChl content (*p* = 0.0273), the available nitrogen could have been used for chlorophyll synthesis, which could explain the differences between the TChl in inoculated plants versus control plants. Our results were consistent with those of Ruíz-Sánchez et al. [53], Naveed et al. [55], and Cohen et al. [56].

In this experiment, we found the highest concentrations of ABA in control plants in the D condition. This shows that stressful conditions trigger a signaling pathway that leads to ABA biosynthesis. The control seedlings in the D condition were pale green, smaller in size (lower aerial and radical biomass), had lower turgidity (the plants had fallen or were low), and showed other symptoms of stress, such as leaf senescence. This is an expected result, according to Salinas-Moreno and González-Hernández [73], who showed that a decrease in the hydric potential of corn leaves increased ABA production. In drought conditions, this process prevents the plant from losing water through transpiration. In turn, this decreases the photosynthesis rate, which leads to the observed loss of color (chlorophyll) and slowed or halted growth. The ABA concentration results (Figure 5) showed that in the WW condition, control plants had a mean of 0.25 μg/gFW ABA, whereas inoculated plants had a mean of 0.05 μg/gFW ABA. In the D condition, control plants showed an increase in the ABA concentration, whereas inoculated plants did not show a similar response. Our results showed that inoculation with PGPR had a negative effect on ABA synthesis in plants. The inoculated plants had a lower stress level. On the other hand, the inoculated plants did not show significant changes in the level of *ZmVP14* expression in both WW and D conditions, except those inoculated with *H. seropedicae*, which showed significant differences (inoculation * drought interaction *p* < 0.0001). It has been reported that *Bacillus subtilis*, strain B26, confers resistance against drought stress in *Brachypodium* and this is linked to the upregulation of expression of several drought-responsive genes and the modulation of the DNA methylation process [74].

The ethylene content was also lower in inoculated plants (*p* = 0.0297) (Figure 6). This could be due to the effects of the ACC deaminase produced by the bacteria, which may mitigate the deleterious effect of ethylene, thereby lowering the stress level in the plant and promoting plant growth [18]. The RWC (Table 1) was higher in inoculated plants in both the WW and D conditions (*p* < 0.0001). It is possible that the integrity of the plasma membrane was better due to the beneficial effects of inoculation, which may have mitigated the damage by the ROS produced in stressful conditions. This was shown by Naveed et al. [55], Cohen et al. [56], and Tiwari et al. [57]. The MDA results (Figure 8) showed that the PGPR-inoculated seedlings had a lower level of damage during drought stress, because in the D condition, control plants showed a significant increase in the MDA level, which is an indicator of the damage caused by lipid peroxidation. The consequences of lipid peroxidation include deterioration in the cellular membrane, deterioration in the selective permeability of the membrane and, finally, cellular disintegration and death. We observed the same effects in the MDA concentrations in the WW condition regardless of inoculation status, demonstrating that inoculation does not affect the MDA in WW conditions. In contrast, in the D condition, there was a clear difference between inoculated plants and control plants. These results may be related to the RWC (Table 1), because less cellular membrane damage would allow a higher water content level inside the cells. Importantly, MDA analysis is used in tolerance tests of corn crops under hydric stress [75], and these results show that in D conditions, the bacteria help plants reduce membrane damage.

Osmolytes synthesis induced by ABA can also help protect plants against drought stress. Notably, osmolytes can decrease the hydric potential of the cell and thereby help it to avoid losing water. Proline is one type of osmolyte, and in this experiment, changes in the ABA concentration (Figure 5) were similar to the changes in the proline concentration (Figure 7) in the D condition. It appeared that non-inoculated plants could not use this physiological tool to maintain the RWC because the control plants were not turgid and, in many cases, had fallen. These results are in agreement with those obtained by Paleg et al. [76] and Ashraf and Foolad [77], who showed that proline synthesis can be seen as a symptom of stress in plants, taking into account their pathway of induction. It has also been reported that plants that were inoculated with PGPR (*Pseudomonas* sp.) bacteria showed a decrease in proline synthesis when they were under water stress [57]. This would be in agreement with our results, that plants inoculated with *A. brasilense* and *H. seropedicae* may have a lower level of stress in the D condition than control plants. Finally, Bogges et al. [78] determined that water stress prevents the oxidation of proline, resulting in higher proline levels. In addition, other researchers working with *Bacillus megaterium* BOFC15 and *Arabidopsis* found that spermidine improves drought tolerance in plants, which was associated with altered levels of ABA [79].

The *ZmVP14* gene expression analysis (Figure 9) showed that in both the WW and D conditions, control plants showed higher *ZmVP14* expression than inoculated plants. Notably, *ZmVP14* expression is induced when the seedlings are under hydric stress. Our data showed that there were differences in inoculated versus non-inoculated plants in terms of genetic regulation of ABA production that resulted in differences in ABA concentration. Our *ZmVP14* expression results agreed with those of Tan et al. [16] and with those of Chernys and Zeevaart [80], who showed that the inhibition of *ZmVP14* affects ABA biosynthesis. Furthermore, the inoculated plants grew well even under conditions of water deficit, in addition the inoculated plants had lower levels of ABA than the control plants. Perhaps because there is some kind of mechanism induced for the presence of bacteria that allows sustaining a good growth and good water ratio in the plant.

Our results are probably in agreement and are explained by the highest RWC, such as that found in References [81,82]. Changes in the distribution of specific fatty acids in the root [82] and polyamines—compounds that regulate the growth of plants, including cadaverine—have been correlated with the growth of the radical system or the mitigation of osmotic stress in some plant species [83].

## 5. Conclusions

Our results show that the inoculation of maize plants with *A. brasilense* or *H. seropedicae* bacteria had important effects on the tolerance of the maize to drought stress. The bacteria had direct effects on physiological, biochemical, and molecular processes that resulted in reduced stress to the plants. The results very clearly show a large positive effect on growth under drought and a reduced stress response (proline, ethylene), and reduced evidence of stress (MDA). Inoculation with these bacteria also greatly affected % total carbon, ABA content, and ethylene content. These results suggest that these bacteria could be used to reduce the effects of drought stress and thereby improve the productivity of maize crops in drought conditions.

## Figures and Tables

**Table 1:** Effect of inoculation on the relative water content (RWC) of plants in well-watered (WW) and drought (D) conditions. Means with common letters are not significantly different (*p* > 0.05). Data are means of six replicates ± standard deviation (SD). The inoculation x drought interaction *p* < 0.0001.

Treatment	RWC (%)	
	WW	D
Control	52.71 ± 2.5 ^c^	45.45 ± 1.17 ^a^
*A. brasilense*	58.81 ± 2.7 ^e^	47.86 ± 1.37 ^b^
*H. seropedicae*	53.93 ± 3.16 ^d^	48.1 ± 1.95 ^b^
